# Comparison of prophylactic response to lithium and valproate in patients with Early Onset Bipolar Disorder

**DOI:** 10.1192/j.eurpsy.2023.1478

**Published:** 2023-07-19

**Authors:** M. Purkayastha MUKHERJEE, G. G, P. Kandasamy, S. S, D. pandian

**Affiliations:** 1 Psychiatry, JIPMER, Puducherry; 2 Psychiatry, Maanas, Salem; 3 Pharmacology, JIPMER, Puducherry, India

## Abstract

**Introduction:**

The clinical presentation and the course and outcome in Early-Onset Bipolar Disorder (EOBD) patients are found to be atypical compared to adult bipolar patients. Lithium and valproate are among the first-line maintenance treatments for bipolar disorder. Because of atypical features, in many patients, valproate is preferred over lithium. However, recent studies have shown that valproate results in more neurocognitive deficits than lithium. There have been very few Indian studies that assessed the prophylactic response to mood stabilizers in patients with early-onset bipolar disorder. BDNF has an important role in neurodevelopment, and it is shown that peripheral levels of BDNF are reduced in early-onset bipolar disorder.

**Objectives:**

To compare the effectiveness of lithium and valproate in attenuating manic, depressive, and mixed episodes in early-onset bipolar disorder.

**Methods:**

This study was an observational (cross-sectional analytical) study conducted in the Affective Disorder clinic of a tertiary care hospital. We have recruited a total of 50 adult patients with a history of early-onset, i.e., onset at <18 years of age and in remission. Patients were divided into two groups based on the mood stabilizer drug they were receiving. There were 25 patients each in the lithium and valproate group. Montreal Cognitive Assessment (MoCA) scale was applied to assess cognitive functions.

**Results:**

The overall functioning was found to be significantly better in the patients receiving lithium than valproate, which was found by higher scores on the Global Assessment of Functioning (GAF) scale.We have found a statistically significant negative correlation between the number of episodes before starting a mood stabilizer and the time to recurrence after starting a mood stabilizer. However, the former cannot predict the latter. The age, educational status of the patient, total duration of illness and number of episodes before starting mood stabilizer correlated significantly with the MoCA score. Of all, the educational status could also predict the patient’s performance on the MoCA scale.

**Conclusions:**

There were no significant differences between lithium and valproate in attenuating further episodes, the frequency of neurocognitive deficits and other adverse effects. Both drugs were equally effective and tolerable. The severity of illness was more in valproate-treated patients, and overall functioning was better in lithium-treated patients. BDNF levels did not correlate with neurocognitive deficits significantly. Future studies comprehensively assessing neurocognitive measures with a larger sample size in the early-onset bipolar disorder population would shed more light on the role of biomarkers in cognition in subjects with early-onset bipolar disorder.

**Disclosure of Interest:**

None Declared

